
JOURNAL INFORMATION
==============================
NLM Title Abbreviation: Sci China Life Sci
Journal ID: Sci China Life Sci
NLM Journal ID: 101529880

Science China. Life Sciences

ISSN: 1674-7305
EISSN: 1869-1889

ARTICLE INFORMATION
==============================
PMCID: PMC7089303
PMID: 23917837
DOI: 10.1007/s11427-013-4525-x
Article ID: 4525
Article version: 1
Subjects: Editorial

Haunted with and hunting for viruses

Gao George Fu gaofu@chinacdc.cn
1 2 3
Wu Ying wuying@im.ac.cn
2
1 grid.198530.6 0000000088032373 Chinese Center for Disease Control and Prevention, Beijing, 102206 China
2 grid.9227.e 0000000119573309 CAS Key Laboratory of Pathogenic Microbiology and Immunology, Institute of Microbiology, Chinese Academy of Sciences, Beijing, 100101 China
3 grid.9227.e 0000000119573309 Research Network of Immunity and Health, Beijing Institutes of Life Science, Chinese Academy of Sciences, Beijing, 100101 China
Electronic publication date: 2013 Aug 7
Print publication date: 2013
Volume: 56
Issue: 8
First page: 675
Last page: 677
Received 2013 Jul 5
Copyright: © The Author(s) 2013
License: Open AccessThis article is distributed under the terms of the Creative Commons Attribution 2.0 International License (https://creativecommons.org/licenses/by/2.0), which permits unrestricted use, distribution, and reproduction in any medium, provided the original work is properly cited.
License URL: https://creativecommons.org/licenses/by/2.0/

issue-copyright-statement: © Science in China Press and Springer-Verlag GmbH 2013

==============================
This article is published with open access at Springerlink.com


References

1 Gao R B Cao B Hu Y W Human infection with a novel avian-origin influenza A (H7N9) virus N Engl J Med 2013 368 1888 1897 10.1056/NEJMoa1304459 23577628
2 Chen Y Liang W Yang S Human infections with the emerging avian influenza A H7N9 virus from wet market poultry: clinical analysis and characterisation of viral genome Lancet 2013 381 1916 1925 10.1016/S0140-6736(13)60903-4 23623390 PMC7134567
3 Li J Yu X F Pu X Y Environmental connections of novel avian-origin H7N9 influenza virus infection and virus adaptation to the human Sci China Life Sci 2013 56 485 492 10.1007/s11427-013-4491-3 23657795
4 Liu D Shi W F Shi Y Origin and diversity of novel avian influenza A H7N9 viruses causing human infection: phylogenetic, structural, and coalescent analyses Lancet 2013 381 1926 1932 10.1016/S0140-6736(13)60938-1 23643111
5 Gao G F Sun Y It is not just AIV: from avian to swine-origin influenza virus Sci China Life Sci 2010 53 151 153 10.1007/s11427-010-0017-4 20596968
6 Zaki A M van Boheemen S Bestebroer T M Isolation of a novel coronavirus from a man with pneumonia in Saudi Arabia N Engl J Med 2012 367 1814 1820 10.1056/NEJMoa1211721 23075143
7 Lu G Liu D SARS-like virus in the Middle East: a truly bat-related coronavirus causing human diseases Protein Cell 2012 3 803 805 10.1007/s13238-012-2811-1 23143870 PMC4875465
8 Lu G Hu Y Wang Q H Molecular basis of novel human coronavirus MERS-CoV bound to its receptor CD26 Nature 2013 10.1038/nature12328 PMC7095341 23831647
9 Wu Y Gao G F Severe fever with thrombocytopenia syndrome virus expands its borders Emerg Microbes Infect 2013 10.1038/emi.2013.36 PMC3697302 26038472
10 Tong S Li Y Rivailler P A distinct lineage of influenza A virus from bats Proc Natl Acad Sci USA 2012 109 4269 4274 10.1073/pnas.1116200109 22371588 PMC3306675
11 Xu B L Liu L C Huang X Y Metagenomic analysis of fever, thrombocytopenia and leukopenia syndrome (FTLS) in Henan Province, China: discovery of a new bunyavirus PLoS Pathog 2011 7 e1002369 10.1371/journal.ppat.1002369 22114553 PMC3219706
12 Chen H L H5N1 avian influenza in China Sci China Ser C-Life Sci 2009 52 419 427 10.1007/s11427-009-0068-6 19471864
13 Liu D Liu Q H Wu L H Website for avian flu information and bioinformatics Sci China Ser C-Life Sci 2009 52 470 473 10.1007/s11427-009-0065-9 19471870
14 Liu D Liu X L Yan J H Interspecies transmission and host restriction of avian H5N1 influenza virus Sci China Ser C-Life Sci 2009 52 428 438 10.1007/s11427-009-0062-z 19471865 PMC7089370
15 Shi Z L Emerging infectious diseases associated with bat viruses Sci China Life Sci 2013 56 678 682 10.1007/s11427-013-4517-x 23917838 PMC7088756
16 Shi Z L Bat and virus Protein Cell 2010 1 109 114 10.1007/s13238-010-0029-7 21203979 PMC4875169
17 Woo P C Lau S K Lam C S Discovery of seven novel mammalian and avian coronaviruses in the genus Deltacoronavirus supports bat coronaviruses as the gene source of Alphacoronavirus and Betacoronavirus and avian coronaviruses as the gene source of Gammacoronavirus and Deltacoronavirus J Virol 2012 86 3995 4008 10.1128/JVI.06540-11 22278237 PMC3302495
18 Woo P C Wang M Lau S K Comparative analysis of twelve genomes of three novel group 2c and group 2d coronaviruses reveals unique group and subgroup features J Virol 2007 81 1574 1585 10.1128/JVI.02182-06 17121802 PMC1797546
19 Ge X Y Li Y Yang X L Metagenomic analysis of viruses from bat fecal samples reveals many novel viruses in insectivorous bats in China J Virol 2012 86 4620 4630 10.1128/JVI.06671-11 22345464 PMC3318625
20 Johnson N Vos A Freuling C Human rabies due to lyssavirus infection of bat origin Vet Microbiol 2010 142 151 159 10.1016/j.vetmic.2010.02.001 20188498
21 Rupprecht C E Turmelle A Kuzmin I V A perspective on lyssavirus emergence and perpetuation Curr Opin Virol 2011 1 662 670 10.1016/j.coviro.2011.10.014 22440925
22 Geng H Y Tan W J A novel human coronavirus: Middle East respiratory syndrome human coronavirus (MERS-CoV) Sci China Life Sci 2013 56 683 687 10.1007/s11427-013-4519-8 23917839 PMC7089429
23 Raj V S Mou H Smits S L Dipeptidyl peptidase 4 is a functional receptor for the emerging human coronavirus-EMC Nature 2013 495 251 254 10.1038/nature12005 23486063 PMC7095326
24 Yang F Jin Q He Y Q The complete genome of Enterovirus 71 China strain Sci China Ser C-Life Sci 2001 44 178 183 10.1007/BF02879323 18726435
25 Yang F Ren L L Xiong Z H Enterovirus 71 outbreak in the People’s Republic of China in 2008 J Clin Microbiol 2009 47 2351 2352 10.1128/JCM.00563-09 19439545 PMC2708525
26 Xie G Y Duan J Z New strategy for virus discovery: viruses identified in human feces in the last decade Sci China Life Sci 2013 56 688 696 10.1007/s11427-013-4516-y 23917840 PMC7089042
27 Zhang X S Liu Y Zhao L An emerging hemorrhagic fever in China caused by a novel bunyavirus SFTSV Sci China Life Sci 2013 56 697 700 10.1007/s11427-013-4518-9 23917841
28 Liu P P Lu H Li S Duck egg drop syndrome virus (DEDSV): an emerging Tembusu-related flavivirus in China Sci China Life Sci 2013 56 701 710 10.1007/s11427-013-4515-z 23917842
29 Liu P P Lu H Li S Genomic and antigenic characterization of the newly emerging Chinese duck egg-drop syndrome flavivirus: genomic comparison with Tembusu and Sitiawan viruses J Gen Virol 2012 93 Pt10 2158 2170 10.1099/vir.0.043554-0 22764316
30 Su J L Li S Hu X D Duck egg-drop syndrome caused by BYD virus, a new Tembusu-related flavivirus PLoS ONE 2011 6 e18106 10.1371/journal.pone.0018106 21455312 PMC3063797
31 Liu M Chen S Y Chen Y H Adapted Tembusu-like virus in chickens and geese in China J Clin Microbiol 2012 50 2807 2809 10.1128/JCM.00655-12 22692734 PMC3421502
32 Tang Y Diao Y Yu C Characterization of a Tembusu virus isolated from naturally infected house sparrows (Passer domesticus) in Northern China Transbound Emerg Dis 2012 10.1111/j.1865-1682.2012.01328.x 22515847
33 Qiu Y Wang Z W Liu Y X Newly discovered insect RNA viruses in China Sci China Life Sci 2013 56 711 714 10.1007/s11427-013-4520-2 23917843
